# Yellow Fever Vaccine–Associated Viscerotropic Disease among Siblings, São Paulo State, Brazil

**DOI:** 10.3201/eid2903.220989

**Published:** 2023-03

**Authors:** Eder Gatti Fernandes, Victor Bertollo Gomes Porto, Patrícia Mouta Nunes de Oliveira, Amaro Nunes Duarte-Neto, Maria de Lourdes de Sousa Maia, Letícia Kegele Lignani, Juliana Silva Nogueira, Gabriellen Vitiello Teixeira, Silvia D’Andretta Iglezias, Roberta Morozetti Blanco, Helena Keico Sato

**Affiliations:** Instituto de Infectologia Emílio Ribas, São Paulo, Brazil (E.G. Fernandes);; Centro de Vigilância Epidemiológica “Prof. Alexandre Vranjac,” São Paulo (E.G. Fernandes, H.K. Sato);; Programa Nacional de Imunizações, Brasilia, Brazil (V.B.G. Porto);; Bio-Manguinhos, Rio de Janeiro, Brazil (P.M.N. de Oliveira, M. de Lourdes de Sousa Maia, L.K. Lignani, G.V. Teixeira);; Faculdade de Medicina da Universidade de São Paulo, São Paulo (A.N. Duarte-Neto);; Instituto Adolfo Lutz, São Paulo (A.N. Duarte-Neto, J.S. Nogueira, S. D'Andretta Iglezias, R.M. Blanco)

**Keywords:** yellow fever vaccine, adverse events, viscerotropic disease, vector-borne infections, São Paolo, Brazil, viruses

## Abstract

We describe 5 cases of yellow fever vaccine–associated viscerotropic disease (YEL-AVD) in 2 familial clusters during the 2017–2018 yellow fever (YF) vaccination campaign in São Paulo state, Brazil. The first case was that of a 40-year-old white man who died of icterohemorrhagic syndrome, which was confirmed to be YEL-AVD by using real-time reverse transcription PCR to detect 17DD YF vaccine in the liver. Ten years previously, his brother died of a clinically similar disease without a confirmed diagnosis 9 days after YF vaccination. The second cluster included 3 of 9 siblings in whom hepatitis developed in the first week after receiving fractionated doses of YF vaccine. Two of them died of hemorrhagic diathesis and renal and respiratory failure, and 17DD-YF vaccine was detected in serum samples from all patients and in the liver in 1 case. Genetic factors might play a substantial role in the incidence of YEL-AVD.

Brazil recently experienced its largest-recorded yellow fever (YF) outbreak in decades; >2,000 human cases and ≈750 deaths occurred during July 2016–June 2018 (*1*). The virus reached the metropolitan areas, where the YF vaccine had not been previously recommended. This situation led to the need for vaccination campaigns in affected areas, which included fractionated doses of the 17DD YF vaccine. In 2017, >5 million persons were vaccinated with the standard dose in the state of São Paulo, Brazil. During January 25–July 3, 2018, the state also used a fractionated-dose vaccine as part of a dose-sparing strategy in 54 municipalities. The fractionated dose consisted of 0.1 mL (one fifth of the standard dose) administered in the subcutaneous tissue to person >2 years of age. This approach was necessary because of insufficient vaccine stock for the entire population at the height of the epidemic. In 2018, a total of 10 million persons were vaccinated; of those, 5.3 million received the standard dose and 4.7 million received the fractionated dose.

During the 2016–2018 YF epidemic, southeastern Brazil was the most affected area; São Paulo state had 559 confirmed cases of YF and 214 deaths attributed to YF (*2*). As a consequence, the YF vaccination coverage rate, which was 5% of the population in 2016, increased to 65% by the end of 2018 in the metropolitan area of the city of São Paulo.

In late 2017, the Health Surveillance Department of the State of São Paulo (Centro de Vigilância Epidemiológica “Prof. Alexandre Vranjac”) received a report of a man in the metropolitan area of São Paulo city who died after YF vaccination. The patient’s brother had died soon after YF vaccination 10 years previously, but cause of death was undetermined. Two months later, the surveillance team received a report from Natividade da Serra in São Paulo state of 3 temporary adverse events associated with YF vaccination among siblings of 1 family that ended in 2 deaths.

The data resulted in a suspicion of YF vaccine–associated viscerotropic disease (YEL-AVD). They were not the first suspected cases of YEL-AVD during the 2017–2018 vaccination campaign. However, the report of 2 familial clusters of severe adverse events following immunization (AEFI) related to YF vaccine in the state of São Paulo triggered a field epidemiologic investigation. In this descriptive study, we describe the epidemiologic investigation of the 2 clusters of YEL-AVD among family members.

## Methods

We used the AEFI surveillance system of São Paulo state, Brazil, to capture information from January 2017–June 2018. The cases reported are level 1 according to Brighton Collaboration Viscerotropic Disease Working Group case definition in terms of the diagnostic certainty (definite or probable YF vaccine–associated causality) (*3*). All case-patients were first-degree relatives (siblings) with YEL-AVD. We also included 1 previous case-patient who had onset of symptoms before 2017 but was related to a current case-patient.

We obtained information regarding the vaccine and clinical and laboratory findings from the official AEFI passive surveillance system database. A field investigation team reviewed medical records. The team also visited the patients’ families to collect clinical and epidemiologic details, in addition to the vaccination status of first-degree relatives, and searched for other suspected cases of AEFI. Last, the team traced the biologic samples for additional and specific laboratory investigations.

We analyzed the available serum and postmortem samples at the Instituto Adolfo Lutz, the central public health laboratory for YF and other notifiable diseases. We extracted the viral RNA from 140 µL of serum by using a QIAamp Viral RNA Minikit (QIAGEN, https://www.qiagen.com) according to the manufacturer’s instructions. We tested the viral genome by using the real-time reverse transcription PCR (RT-PCR) protocol as described by Domingo et al. (*4*). Positive samples were submitted to a second RT-PCR reaction, according to Bae et al. (*5*). This reaction can detect 17DD viral genome but cannot amplify viral genome from wild-type strains circulating in the Americas. We considered samples that tested positive by both RT-PCR protocols positive for vaccine-associated virus. We isolated the YF virus from serum samples using cultured cells of *Aedes albopictus*, clone C6/36, and performed viral identification using indirect immunofluorescence assay with polyclonal YF antibodies and conjugated antimouse immunoglobulins. We used the US Centers for Disease Control and Prevention MAC-ELISA protocol for detecting IgM (*6*). The postmortem samples were analyzed by 2 expert pathologists in infectious diseases and YF pathology (S.D’A.I. and A.N.D.N.). We performed immunohistochemistry reactions to detect in situ YF virus antigens using a polyclonal wild YF virus strain primary antibody (*7*). This study was approved by the Research Ethics Committee of the Health Department of the Municipality of São Paulo (approval no. CAAE 37233620.5.0000.0086).

## Results

### Cluster 1

**Figure 1:**
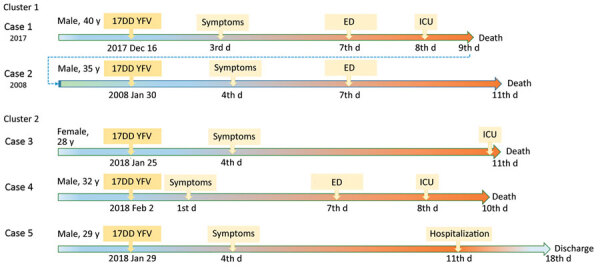
Timelines of reported cases of YFV–associated viscerotropic disease after 17DD vaccination during yellow fever epidemic, São Paulo state, Brazil, 2017–2018. Cases 1–2 were brothers and received standard doses. Cases 3–5 are siblings and received fractionated doses. ED, emergency department; ICU, intensive care unit; YFV, yellow fever vaccine.

Case 1 was that of a 40-year-old white man who received the first dose of 17DD YF vaccine (lot 174VFC056Z) on December 16, 2017 (Figure 1). No simultaneous vaccines were administered. On day 3 after vaccination, headache, malaise, and fever developed, and he took analgesics. After a day of clinical improvement, his symptoms returned, along with a high fever, nausea, and vomiting, for the next 3 days. On day 7 after vaccination, he was admitted to the emergency department for diffuse abdominal pain and hypotension. Initial laboratory tests revealed only thrombocytopenia (platelets 51,000/mm^3^ [reference range 150,000–450,000/mm^3^]). On day 8 after vaccination, he had fever, jaundice, conjunctival hyperemia, peripheral edema, hypotension, dyspnea, tachycardia, and oliguria. The patient was transferred to a tertiary-care center in critical condition experiencing respiratory distress, shock, and metabolic acidosis (arterial blood gas pH 6.8, bicarbonate 9 mmol/L, carbon dioxide partial pressure 58 mm Hg, and partial pressure of oxygen 50 mm Hg). He was started on mechanical ventilation and was administered antibiotics (piperacillin and tazobactam), fluids, and vasoactive drugs. Chest radiography revealed bilateral pulmonary congestion in the middle-upper lobes. Laboratory tests revealed increased total leukocyte count with left shift (total leukocyte count 34,700 cells/mm^3^; promyelocytes 2%, myelocytes 4%, metamyelocytes 8%, rods 34%, and neutrophils 42%), as well as increased serum levels of creatinine, urea, creatine phosphokinase, liver enzymes, and bilirubin. His platelets dropped to 38,000/mm^3^, and the international normalized ratio (INR) increased (Table). The serum level of C-reactive protein increased (20.66 mg/dL). The patient died on December 25, 2017, on day 9 after vaccination (Figure 1), day 6 after illness onset.

**Table:** Clinical findings of cases of adverse events after YF vaccination in siblings from 2 familial clusters, São Paulo state, Brazil, 2017–2018*

Case information	Cluster 1			Cluster 2		
	Case 1	Case 2		Case 3	Case 4	Case 5
Patient age, y/sex	40/M	35/M		28/F	32/M	29/M
Patient race	White	White		White	White	White
Kind of YF vaccine	Standard	Standard		Fractionated	Fractionated	Fractionated
Days from vaccination to symptom onset	3	4		4	1	4
Symptoms	Fever, headache, vomiting, abdominal pain, jaundice, bleeding	Fever, myalgia, vomiting, diarrhea, jaundice, seizures		Fever, myalgia, abdominal pain, dysuria, vomiting, bleeding	Fever, myalgia, abdominal pain, jaundice, bleeding	Fever, vomiting, myalgia, headache
Platelets, per μL†	38,000	29,000		23,000	29,000	75,000
Aspartate transaminase, U/L†	366	2,254		769	1,930	137
Alanine aminotransferase, U/L	166	617		180	503	122
International normalized ratio†	1.94	1.8		1.9	2.0	2.0
Total bilirubin, mg/dL†	8.5	8.5		6.5	7.6	0.6
Creatinine, mg/dL†	6.7	5.9		2.9	5.9	1.4
Creatinine phosphokinase†	428	1,061		13,080	2,770	514
Outcome	Died	Died		Died	Died	Recovered
RT-PCR for YF vaccine in blood	Positive, 9 DAV	Positive, 10 DAV		Positive, 11 DAV	Positive, 10 DAV	Positive, 14 DAV
Viral isolation in serum sample >7 DAV	Positive, 9 DAV	ND		ND	ND	ND
YF IgM	Detected, 9 DAV	ND		ND	Detected, 10 DAV	Negative, 14 DAV
Liver histopathological pattern at autopsy	Typical‡	ND		ND	Typical‡	ND
YF immunohistochemistry in hepatic tissue	Positive	NA		NA	Positive	NA
RT-PCR for YF in liver	ND	ND		ND	Positive	ND

*DAV, days after vaccination; NA, not applicable; ND, not done; RT-PCR, reverse transcription PCR; YF, yellow fever. †Most altered result. ‡The liver exhibited discrete microvesicular fatty change and mixed sinusoidal inflammatory infiltrate, with no evidence of hepatocellular necrosis in the sample.

**Figure 2:**
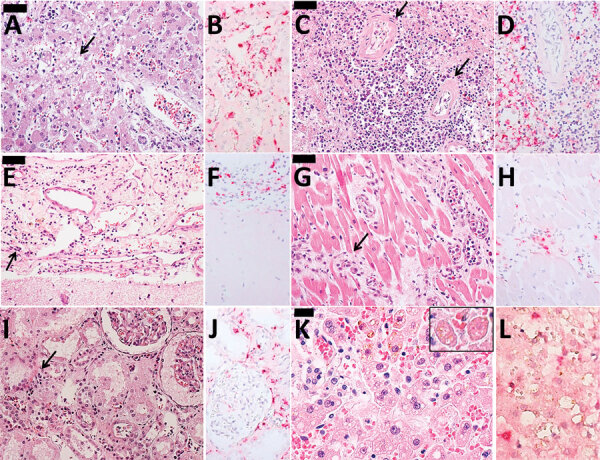
Histopathological findings in 2 case-patients with fatal reaction to 17DD yellow fever (YF) vaccine, São Paolo state, Brazil, 2017–2018. A, B) Liver tissue from case 1: A) steatotic hepatitis (arrow) with scarce inflammatory reaction and rare apoptotic bodies; B) immunostaining for YF antigens in Kuppfer cells and inflammatory cells in the portal tract. C, D) Spleen tissue from case 1: C) lymphoid hypoplasia (arrows); D) YF antigens in cells located on the white pulp and red pulp. E, F) Meningeal tissue from case 1: E) mononuclear meningoencephalitis (arrow); F) YF antigens detected in the cytoplasm of meningeal inflammatory cells. G, H) Cardiac tissue from case 1: G) mononuclear interstitial myocarditis (arrow); H) positive YF antigens detected in the inflammatory cells; I, J) Kidney tissue from case 1: I) acute tubular necrosis and interstitial nephritis (arrow); J) detectable YF antigens in the inflammatory cells. K ,L) Liver tissue from case 2: K) steatotic hepatitis with rare apoptotic bodies (inset); L) scarce immunodetection of YF antigens in the Kuppfer cells. Panels A, C, D, E, G, I, and K are hematoxylin and eosin stained; B, D, F, H, J, and L are immunohistochemistry with alkaline phosphatase conjugated polymer, using a mouse polyclonal YF antibody directed to wild strain (Instituto Adolfo Lutz, São Paolo, Brazil). Scale bars in panels A, C, E, and G indicate 50 µm and in panel K indicates 20 µm. Original magnification ×400.

Autopsy results (Figure 2, panels A–J) revealed microvesicular steatosis with discrete and mixed portal and sinusoidal inflammatory infiltrates in the liver; acute tubular necrosis and interstitial nephritis in the kidneys; edema and interstitial mononuclear infiltrates in the myocardium; pulmonary hemorrhages; mild cerebral edema; and hypoplasia of the white pulp of the spleen. Immunohistochemistry detected YF antigens in the Kupffer cells, rare hepatocytes, and in inflammatory cells in the liver, kidneys, heart, and spleen. The same 17DD strain was identified by RT-PCR in a serum sample collected on December 25 (day 9 after vaccination), and the virus was isolated after inoculation in C6/36 cells.

Case 2 was that of a 35-year-old man who received the first dose of 17DD YF vaccine (lot information unavailable) on January 30, 2008 (Figure 1), because he wished to travel to YF-endemic areas of Brazil. On February 3, 2008 (day 4 after vaccination), he began experiencing fever (38.5°C) and myalgia. The symptoms progressively worsened; 3 days later, he was hospitalized with vomiting, diarrhea, petechiae, and jaundice. He began experiencing oliguria, mental confusion, and generalized clonic-tonic convulsions on day 7 after vaccination and was transferred to a tertiary-care center. Laboratory tests revealed high INR (1.37), increased serum direct and indirect bilirubin levels (8.49 and 7.22 mg/dL), elevated liver enzymes (aspartate aminotransferase [AST] 492 U/L and alanine aminotransferase [ALT] 189 U/L), thrombocytopenia (platelets 52,000/mm^3^), leukocytosis (24,300 cells/mm^3^) with an increase in neutrophil proportion (6% rods and 81% segmented cells), and acute renal failure (creatinine 4.8 mg/dL, urea 131 mg/dL). The patient was started on mechanical ventilation and administered fluids, platelet transfusions, and ceftriaxone for possible acute meningitis. However, the patient experienced progressive multiple organ dysfunction with worsening liver and renal functions and coagulopathy (Table). The patient died on day 11 after vaccination (Figure 1), day 9 after illness onset.

After the death of the patient in case 1, the surveillance team traced and recovered a serum sample from case 2, which was collected on February 9, 2008 (day 10 after vaccination) and stored at −20°C in the Instituto Adolfo Lutz for 10 years. This sample was sent to Biomanguinhos/Fiocruz (Rio de Janeiro, Brazil) for further analysis, which revealed detectable RNA of 17DD YF with a low viral load. Patients in both cases had 2 siblings and parents who were vaccinated during the YF vaccine campaigns in the state of São Paulo. No AEFI was reported among their relatives.

### Cluster 2

Case 3 was that of a 28-year-old white woman who received the first dose of 17DD YF vaccine (fractionated dose, lot 175VFC064Z) on January 25, 2018 (Figure 1). On day 4 after vaccination, she began experiencing abdominal pain, fever (38°C), vomiting, dysuria, and generalized myalgia. She was prescribed analgesics. On day 11 after vaccination, she returned for medical assistance because her condition had worsened and included tachycardia and tachypnea. Laboratory tests revealed leukocyte count of 19,370 cells/mm^3^ with 77% neutrophils and 2% rods; hemoglobin 14 g/dL and hematocrit 40.4%; thrombocytopenia (platelets 29,000/mm^3^); acute renal failure (creatinine 2.92 U/dL, urea 153 U/dL); and elevated serum liver enzymes (AST 464 U/L and ALT 137 U/L) and creatine phosphokinase (CPK) (99 U/L). Urinalysis revealed proteinuria, leukocyturia, and hematuria. The first differential diagnosis was sepsis caused by urinary tract infection, and the patient was prescribed ceftriaxone. She was transferred to a tertiary-care center for respiratory distress, hypoglycemia, oliguria, metabolic acidosis (pH 7.07, bicarbonate 10.3 mmol/L), and progressive shock. In the intensive care unit, she was started on mechanical ventilation and cardiovascular support, but her condition deteriorated. The leukocyte count increased to 31,500 cells/mm^3^ with 88% neutrophils, 5% rods, and 5% lymphocytes, as well as a serum C-reactive protein level of 90.4 mg/dL. The levels of platelets, INR, CPK, and liver function parameters worsened (Table). The patient experienced refractory acidosis and died the same day (Figure 1).

Case 4 was that of a 32-year-old white man who was the brother of the patient in case 3. He received the first dose of 17DD YF vaccine (fractionated dose, lot 173VFA013Z) on February 2, 2018 (Figure 1). On day 1 after vaccination, he began experiencing malaise, myalgia, chest pain, epigastric pain, and fever (39.3°C). He sought medical care at the local ED and was prescribed analgesics. On February 9, 2018 (day 7 after vaccination) he again sought medical care; examination revealed tachycardia (130 bpm), thrombocytopenia (platelets 80,000/mm^3^), proteinuria, leukocyturia, and hematuria. He was prescribed fluid therapy, antipyretics, and ciprofloxacin and was discharged. On the 8th day of illness (February 10, 2018), he returned to the ED with jaundice, dyspnea, hypotension, and a capillary blood glucose level of 63 mg/dL. At that point, laboratory analysis revealed renal insufficiency (creatinine 3.1 U/dL, urea 82 U/dL); increased liver enzyme levels (AST 214 U/L, ALT 95 U/L), increased serum (6.7 mg/dL) and direct (3.12 mg/dL) bilirubin; and increased INR 1.88. CPK level was unremarkable (194 U/L). The patient was treated with fluids and intravenous glucose and was transferred to a tertiary-care center. In the intensive care unit, the patient experienced hematuria, oliguria, agitation, mental confusion, metabolic acidosis (pH 7.08, bicarbonate 15.2 mmol/L), shock, and hypoxemia. He was prescribed mechanical ventilation, ceftriaxone for possible urinary sepsis, intravenous glucose, fresh frozen plasma, cryoprecipitate, hydrocortisone, vasoactive drugs, and hemodialysis. His medical condition continued to deteriorate despite supportive therapy, and multiple organ failure developed (Table). The patient died on day 10 after vaccination (Figure 1), day 9 after illness onset. A postmortem liver sample was collected, and histopathological analysis (Figure 1, panels K, L) revealed midzonal microvesicular steatosis with scattered acidophilic degeneration of hepatocytes (Councilman bodies) and discrete periportal inflammatory reaction, congestion, and cholestasis. Immunocytochemistry detected YF virus antigens in the Kupffer cells and mesenchymal cells. RT-PCR detected RNA of 17DD YF in the hepatic tissue.

Case 5 was that of a 29-year-old white man who was a brother of the patients in cases 3 and 4. He received the first dose of 17DD YF vaccine (fractionated dose, lot 173VFA013Z) on January 29, 2018 (Figure 1). On day 4 after vaccination, he began experiencing nausea, vomiting, generalized myalgia, fever, and headache. On February 7, he sought medical care; the first differential diagnosis was urinary tract infection. Initial laboratory tests revealed leukocyte count of 7,000 cells/mm^3^ with 80% neutrophils and 11% lymphocytes, hemoglobin 14.8 g/dL, hematocrit 44%, and platelets 92,000/mm^3^. Urinalysis revealed hematuria and leukocyturia. The patient was discharged with antipyretics and oral ciprofloxacin. The next day, he began experiencing diffuse abdominal pain and returned to an ED. After normal abdominal ultrasonography, he was administered ﬂuid therapy and antiemetics and discharged. On February 9, 2008, he was admitted to the hospital with the same symptoms, as well as mental confusion and fever. His leukocyte count increased to 22,700/mm^3^ and the platelet count dropped to 75,000/mm^3^, and other test results showed mild acute renal injury (creatinine 1.4 U/dL) and hepatitis (AST 137 U/L, ALT 121 U/L, and INR 2.04). The patient responded well to fluid therapy and antibiotics, and the laboratory parameters returned to normal levels by day 16 after vaccination (Figure 1).

The patients in cases 3–5 were 3 of 11 siblings. Their parents died years before the events reported in this study and their YF vaccination status was unknown. Of the 11 siblings, 9 were vaccinated for YF for the first time during the 2017–2018 campaign. No other AEFI were reported.

The patients in these cases had no recent history of travel, acute medical conditions, or other vaccinations within the previous month. The patient in case 3 was obese, but none of the other patients had relevant medical conditions. In cases 1 and 4, YF IgM was detected using MAC-ELISA. Serum samples from all patients had detectable RNA of 17DD YF strain; samples were collected on the day of death, except in case 5, in which the sample was collected day 14 after vaccination. During hospitalization, all patients had negative results on serologic tests for dengue, leptospirosis, hepatitis B core protein, hepatitis B surface antigen, hepatitis A virus, and HIV, as well as blood and urine cultures.

## Discussion

We describe 2 clusters of siblings with YEL-AVD. The clinical characteristics of all the cases match those of YEL-AVD previously described (*8*–*12*). The symptoms began within the first week after vaccination (range 1–4 days); the initial symptoms were nonspecific (fever, headache, myalgia, nausea, and vomiting) and evolved alongside laboratory abnormalities (thrombocytopenia and elevation of serum total bilirubin, hepatic transaminases, and creatinine levels). The patients who died experienced hypotension and hemorrhage, as well as acute renal and respiratory failure and coagulopathy.

In each cluster, at least 1 case was confirmed (*3*,*13*). In case 1, YF was detected in serum >7 days after vaccination with a typical histopathological pattern and in the liver by immunohistochemistry. Case 4 (cluster 2) included a typical histopathological pattern and positive RT-PCR result for YF in the liver. The other cases were characterized as suspected cases because of their clinical characteristics and lack of available histopathologic details.

Considering the Brighton Collaboration criteria for case definition (*3*), 4 patients (cases 1–4) met >3 major criteria: hepatic abnormalities (total bilirubin >1.5 times the upper limit of normal [ULN] or ALT or AST >3 times ULN), renal abnormalities (creatinine >1.5 times ULN), musculoskeletal abnormalities (CPK >5 times ULN), respiratory abnormalities (oxygen saturation <88% on room air or need for mechanical ventilation), platelet count <100,000/μL, and coagulopathy (INR>1.5). Those patients were classified as level 1 diagnostic certainty according to the case definition of viscerotropic disease (*3*). Case 5 satisfied 2 major criteria (platelet count 75,000/μL and INR 2) with level 2 diagnostic certainty for viscerotropic disease (*3*). However, case 5 had YF 17DD viral RNA in blood samples 14 days after vaccination, which qualifies for diagnosis of a viscerotropic disease and definite yellow fever vaccine–associated causality, according to the Brighton Collaboration (*3*).

Case 2 was identified retrospectively. His clinical manifestations were typical and similar to those of his sibling (case 1). Despite the positive RT-PCR test for YFVV, the viral load was low, and viral isolation was not possible. However, this result does not invalidate the discovery. The serum sample was stored for >10 years, which could have compromised the test to identify the virus.

Ours is not the first report of a viscerotropic disease among siblings in Brazil. A 19-year-old woman experienced YEL-AVD and died; her 12-year-old sister also experienced a severe adverse event after 17DD YF vaccination, which suggested YEL-AVD, but she recovered (*14*). Two siblings 30 and 34 years of age with diagnoses of adrenal insufficiency (Addison’s disease) who were receiving physiologic doses of cortisone died of probable YEL-AVD (*15*).

Among the AEFI cases during the 2017–2018 YF vaccination campaign, cases 3 and 5 (cluster 2) received vaccines from the same lot. Case 4 (cluster 2) and case 1 (cluster 1) received vaccines from different lots. The same lots were distributed to >50 cities and were administered to hundreds of thousands of other persons without any reported serious adverse events (data not shown). No changes in the manufacturing methods of the 17DD vaccine in Brazil could account for the adverse events. Genetic mutations in the YF virus do not seem to be the cause of the adverse reactions because the vaccine virus in the previously confirmed YEL-AVD cases revealed no substantial mutation from the original lot (*12*,*16*,*17*). Furthermore, the YF virus isolates recovered from fatal cases of YEL-AVD revealed no reversion to virulence in animal models (*16*). The occurrence of adverse events might have been related to individual, genetically determined host factors, which is strengthened by the incidence of cases among siblings.

The cases we report strengthen the hypothesis of host genetic predisposition to YEL-AVD. Impaired immunologic profiles have suggested that a robust adaptive immune response with abnormalities in the innate immune system might be involved in the development of YEL-AEFI (*14*,*18*–*21*). The genomic signatures correlated with the immune response to 17DD YF, revealing molecular events observed in the innate immunity against viruses (*22*). Molecules involved in the innate sensing of viruses, such as cytoplasmic receptors of 2,5′-OAS (oligoadenylate synthetase) family members 1, 2, 3 and L, TLR7 (toll-like receptor 7), MDA-5 (melanoma differentiation-associated), and RIG-I (retinoic acid-inducible I), as well as transcription factors that regulate type I interferons (IRF7 [interferon regulatory factor 7] and STAT1 [signal transducer and activator of transcription 1]), are induced (*22*). Genetic variations could result in an impairment of the innate immune response involved in the direct control of viral replication or in mediating viral clearance. The reduced expression of CCR5 (C-C chemokine receptor type 5) because of polymorphism might impair the responsiveness of cells to ligands such as RANTES (Regulated upon Activation, Normal T Cell Expressed and Presumably Secreted) (*20*). Such a breakdown could impair the migration of monocytes to tissue sites of infection with a milder innate response that fails to limit early viral replication (*20*). An investigated fatal case of YEL-AVD revealed genetic variations in the OAS genes that encode essential proteins involved in the innate immune response to viral infections (*17*).

The 17DD YF virus promotes the induction of critical type I interferon (IFN) mediators, such as STAT1 and IRF7 in humans, which lead to the expression of IFN-stimulated genes with antiviral properties, thus effectively mounting an antiviral response in infected and surrounding cells (*22*–*25*). Hernandez et al. (*26*) revealed IFN-αβ receptor 1 deficiency in a 14-year-old girl in whom YEL-AVD was diagnosed without any known risk factors for the disease. In this case, a single-gene inborn error of innate immunity, specifically that of type I IFN cell-intrinsic immunity, was related to the severe adverse reactions to the YF vaccine (*26*). In another study, a case-patient with YEL-AVD had a complete recessive IFN-αβ receptor 2 deficiency (*14*). The patient in case 5 in that study, the only one who survived, underwent exome sequence analysis with other ongoing studies; however, no inborn errors of type I IFN or autoantibodies against IFN I were identified (*14*).

In this study, the siblings in cluster 1 received standard doses of 17DD YF vaccine, whereas the siblings in cluster 2 received fractionated doses. Clinical trials have identified similar viremia and immunogenic responses between the doses. No serious adverse events were identified in these studies, although the sample size was too small for a safety evaluation (*27*–*32*).

Our findings strongly suggest that genetic predispositions in innate immune responses are related to the occurrence of YEL-AVD disease. Future investigations into genetic predispositions to YEL-AVD are warranted. Obtaining a thorough medical history, particularly regarding severe reactions to the vaccine in family members, is advisable before administering the 17DD YF vaccine.
